# Estimation of spatial and temporal variability of pasture growth and digestibility in grazing rotations coupling unmanned aerial vehicle (UAV) with crop simulation models

**DOI:** 10.1371/journal.pone.0212773

**Published:** 2019-03-13

**Authors:** Juan R. Insua, Santiago A. Utsumi, Bruno Basso

**Affiliations:** 1 Facultad de Ciencias Agrarias, Universidad Nacional de Mar del Plata, Balcarce, Buenos Aires, Argentina; 2 Consejo Nacional de Investigaciones Científicas y Técnicas (CONICET), Buenos Aires, Argentina; 3 Department of Animal Science, Michigan State University, East Lansing, Michigan, United States of America; 4 W.K. Kellogg Biological Station, Michigan State University, Hickory Corners, Michigan, United States of America; 5 Department of Earth and Environmental Sciences, Michigan State University, East Lansing, Michigan, United States of America; University of Illinois, UNITED STATES

## Abstract

Systematic monitoring of pasture quantity and quality is important to match the herd forage demand (pasture removal by grazing or harvest) to the supply of forage with adequate nutritive value. The aim of this research was to monitor, assess and manage changes in pasture growth, morphology and digestibility by integrating information from an Unmanned Aerial Vehicle (UAV) and two process-based models. The first model, Systems Approach to Land Use Sustainability (SALUS), is a process-based crop growth model used to predict pasture regrowth based on soil, climate, and management data. The second model, Morphogenetic and Digestibility of Pasture (MDP), uses paddock-scale values of herbage mass as input to predict leaf morphogenesis and forage nutritive value. Two field experiments were carried out on tall fescue- and ryegrass-based pastures under rotational grazing with lactating dairy cattle. The first experiment was conducted at plot scale and was used to calibrate the UAV and to test models. The second experiment was conducted at field scale and was used to test the UAV’s ability to predict pasture biomass under grazing rotation. The Normalized Difference Vegetation Index (NDVI) calculated from the UAV’s multispectral reflectance (n = 72) was strongly correlated (p < 0.001) to plot measurements of pasture biomass (R^2^ = 0.80) within the range of ~226 and 4208 kg DM ha^-1^. Moreover, there was no difference (root mean square error, RMSE < 500 kg DM ha^-1^) between biomass estimations by the UAV (1971±350 kg ha^-1^) and two conventional methods used as control, the C-Dax proximal sensor (2073±636 kg ha^-1^) and ruler (2017±530 kg ha^-1^). The UAV approach was capable of mapping at high resolution (6 cm) the spatial variability of pasture (16 ha). The integrated UAV-modeling approach properly predicted spatial and temporal changes in pasture biomass (RMSE = 509 kg DM ha^-1^, CCC = 0.94), leaf length (RMSE = 6.2 cm, CCC = 0.62), leaf stage (RMSE = 0.7 leaves, CCC = 0.65), neutral detergent fiber (RMSE = 3%, CCC = 0.71), digestibility of neutral detergent fiber (RMSE = 8%, CCC = 0.92) and digestibility of dry matter (RMSE = 5%, CCC = 0.93) with reasonable precision and accuracy. These findings therefore suggest potential for the present UAV-modeling approach for use as decision support tool to allocate animals based on spatially and temporally explicit predictions of pasture biomass and nutritive value.

## Introduction

In most livestock systems, animal feed represents the highest proportion of variable costs. Therefore, a general aim for most grazing-based animal production systems is to maximize profitability by increasing the amount of homegrown forages converted into animal product (meat or milk). Livestock grazing-based systems are required to provide a large quantity of forage of high-nutritive value in the most efficient and cost-effective way. Frequent monitoring of pasture cover is one way to schedule grazing rotations and to allocate forage according to the herd forage demand (pasture removal by grazing or harvest). A proactive approach to allocate pasture forage to animals must consider grazing management as a set of dynamic decisions that take into account the temporal and spatial variation of pasture growth associated mainly to weather, soil nutrients and grazing management factors. However, this approach can be time consuming and requires adequate methods and techniques to systematically monitor changes in pasture cover [1].

Recent advances in sensor technology [2] have allowed the development of lightweight multi-spectral cameras with remote sensors suitable for mounting on unmanned aerial vehicles (UAV) for a variety of purposes related to plant monitoring. This novel UAV-based technology offers the opportunity to use high-resolution (< 1 m) spectral data collected over large areas for calculation of vegetation index that can be interpreted in units of dry matter (DM) biomass, sward height, or nutrient composition. Although UAV-mounted sensors have been used in crop production to measure spatial variability of crops, including yield [3] or distance between plants quickly and accurately [4], they have not been used to estimate and monitor changes of grazed pasture both in space and time.

Practical evidence from on-farm situations indicates that the efficiency of most grazing systems depends largely on the farmer’s ability to accurately track timely changes of pasture biomass both within and across grazed paddocks [5]. However, the accurate monitoring of changes in pasture cover is usually a challenging task. Pasture growth typically exhibits short-term variation in relation to meteorological conditions like temperature, radiation and rainfall, which in turn interact with changes in leaf area associated to grazing pressure and residual pasture cover [6, 7]. A possible solution to accurately estimate both spatially and temporally variations of pasture growth is to integrate remotely sensed multispectral data into biophysical models that can estimate plant growth in relation to growing conditions [8]. In this sense, biophysical simulation models may represent an important complementary tool to field measurements, thereby providing predictions to proactively guide grazing management recommendations [9].

The aim of this research was to monitor and model changes in pasture growth, morphology and digestibility of grazed pastures by integrating site-specific information from UAV and process-based models. We hypothesize that spatial and temporal variations of pasture spectral reflectance can be used in conjunction with results from crop simulation models to accurately monitor and predict changes in pasture growth and nutritive value, and to guide grazing management based on the observed spatial variations. To test this hypothesis, two field experiments were conducted at plot and at field and farm scales on previously established ryegrass- and tall fescue-dominated pasture grazed by lactating dairy cows.

## Materials and methods

### Study site

The study was conducted during spring-summer of 2016 using sixteen 1-ha paddocks of robotic and grazing dairy farm at the Michigan State University’s W.K. Kellogg Biological Station (KBS), Hickory Corners, MI, USA (42°25′N, 85°22′W, 291 m.a.s.l.). The present field study did not involve any use of endangered or protected plant or animal species. The KBS dairy farm included two pre-established grass-based pastures used for rotational grazing with lactating Holstein cows: a tall fescue-based pasture (referred hereafter as Fescue) that consisted of tall fescue (*Festuca arundinacea* Schreb.), orchardgrass (*Dactylis glomerata*), red clover (*Trifolium pretense*) and alfalfa (*Medicago sativa*), and, a ryegrass-based pasture (referred hereafter as Ryegrass) that consisted of perennial ryegrass (*Lolium perenne*) and white clover (*Trifolium repens*). The proportions of legumes were 15% and 40% for Fescue and Ryegrass, respectively. Soils at the dairy farm are well-drained typic hapludalfs with a sandy loam layer over the first 30 cm and a deep profile with moderate water-holding capacity (range: 143 to 223 mm). The soil analysis of top A horizon (0.018 m) was as follows, organic matter content of 22 g kg^-1^, pH 6.4 and P content of 34 mg kg^-1^. Mean annual air temperature at the site is 9.4°C, varying from 21.9°C in July to -4.5°C in January. Mean annual precipitation is 962 mm, 50% of which falls from May to September.

### Integrating UAV and crop model

**Fig 1:**
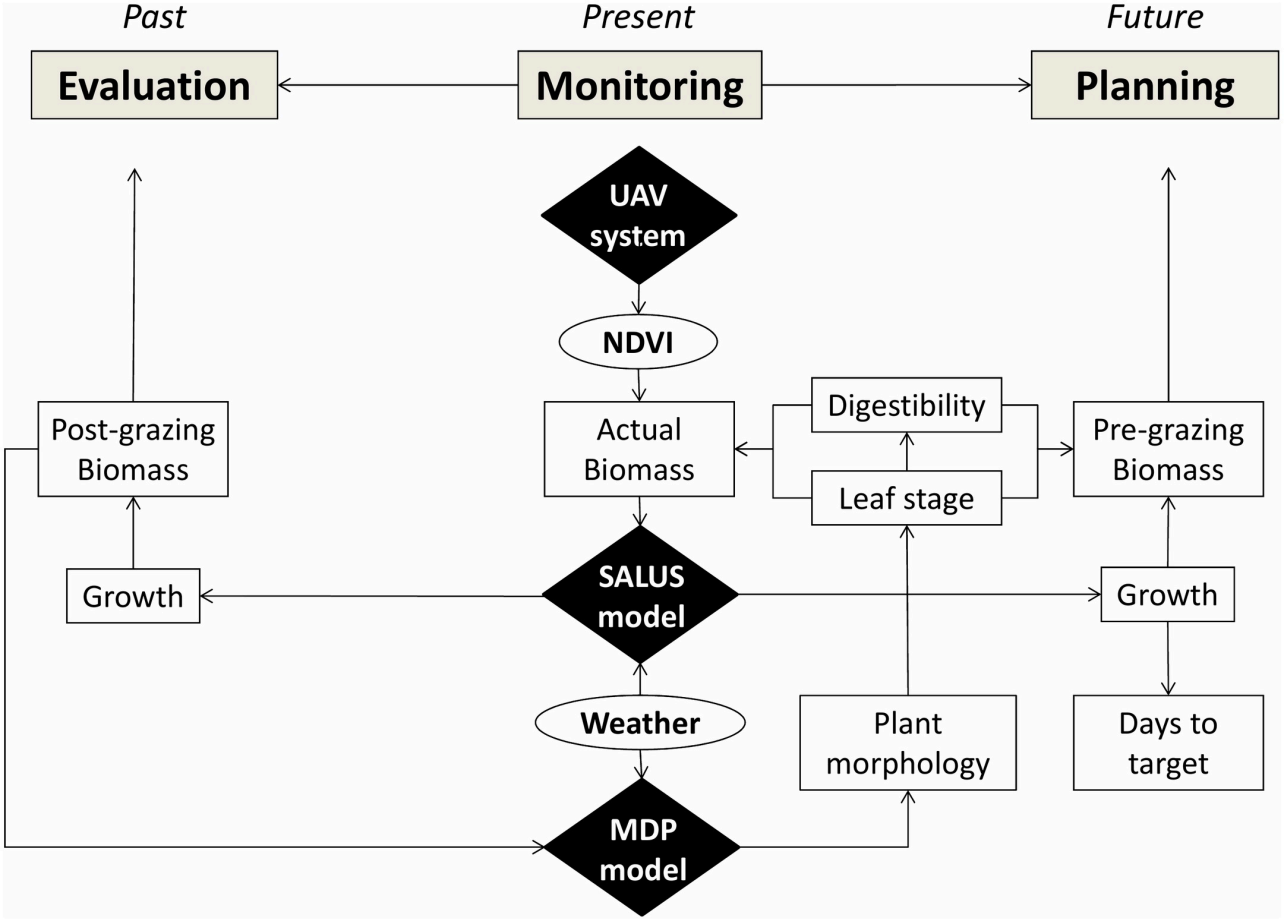
Schematic representation of the integration of a UAV-modeling system.

The experimental approach (Fig 1) integrated the use of an UAV for estimation of actual pasture biomass (kg DM ha^-1^) at paddock scale, and, two process-based models. The first model, Systems Approach to Land Use Sustainability—SALUS, is a process-based crop growth model used to predict pasture regrowth in relation to soil, climate, and management data. The second model, Morphogenetic and Digestibility of Pasture—MDP, uses the estimated pasture biomass to predict temporal changes in leaf stage and digestibility of pasture at sward level [10]. Finally, modeled output data included UAV-derived pasture maps and paddock-scale predictions both of next grazing date and resting length for reaching pre-grazing pasture cover targets, common practice in dairy farms that is usually defined by the use of a predefined grazing wedge.

### Biomass monitoring by UAV

The method used to estimate pasture biomass (kg DM ha^-1^) by the UAV included the following three steps: extraction of geospatial pasture reflectance with UAV-mounted sensor, calculation of Normalized Difference Vegetation Index (NDVI) from pasture reflectance data, and development of the calibration equation to convert NDVI values into kg DM ha^-1^ of pasture. Briefly, multispectral imaging and LIDAR (Light Detection and Ranging) sensors were mounted on a Microdrone md4-1000 UAV to measure canopy reflectance at high spatial resolution (every 6 cm). Flyovers were conducted at ~100 m elevation. All digital captures were then revised, stitched and processed using Pix4DMapper (Pix4D, Lausanne, Switzerland) and ArcGIS software (ESRI, Redlands, CA, USA). Finally, the NDVI was calculated from the acquired geospatial reflectance raster maps, as follows:

$$NDVI=\left({NIR}_{780}-{RED}_{660}\right)/\left({\;NIR}_{780}+{RED}_{660}\right)$$ (1)

[11]

where *RED*_*660*_ and *NIR*_*780*_ are the spectral reflectance in the red (660 nm) and near infrared radiation (780 nm) band, respectively. This NDVI data were then converted to pasture biomass (kg DM ha^-1^) according to the following calibration equation:

$${UAV}_{i}=\alpha e^{\beta \;{NDVI}_{i}}$$ (2)

where *UAVi* is the UAV-derived pasture biomass (kg DM ha^-1^) at paddock scale (*i* = 1-*n*), *α* and *β* are equation parameters determined during the UAV calibration in experiment 1. Values of UAV_*i*_ were used as the input pasture biomass for SALUS crop model.

### Modeling pasture growth using SALUS model

SALUS model is a functional crop simulation model designed to simulate and predicts the effects of the interactions between soil, climate, genetics and management on crop growth and yield, and on environmental outcome. SALUS has a complex and simple modeling approach depending on the type of crops and applications. SALUS has been used in several applied and tested in various contexts (e.g. [12–14]). For our approach, SALUS was used purely as a pasture growth simulator by parameterizing for the physiology of pasture species and the soil type. A companion validation dataset is presented as supplementary material (S1 Table) and is part of the previously published modeling work by [15]. Briefly, the SALUS model [16–18] estimated aboveground growth (kg DM ha^-1^d^-1^) from the product of radiation use efficiency (RUE) and the intercepted photosynthetically active radiation (PAR) that is related to the actual leaf area index (LAI). Soil properties (bulk density and soil texture), crop species description, agronomic management and daily weather data (solar radiation, temperature and precipitation) are required inputs of the model. Water and N stress factors are calculated daily as rate of actual vs potential.

### Linking UAV images with SALUS simulations

We connected the UAV-derived pasture data and SALUS pasture growth simulations “in series.” The predicted daily pasture growth rate by SALUS were exported to a spreadsheet for further computation together with the UAV-derived pasture biomass data and on-farm grazing schedule (i.e. days of regrowth for each paddock).

From UAV-derived pasture biomass (UAV_*i*_, kg DM ha^-1^) data, we also calculated for each paddock (*i* = 1-*n*) the residual pasture biomass (post-grazing kg DM ha^-1^) left by cows in the last grazing event, as follows:

$${Post\;grazing}_{i}={UAV}_{i}-{Growth\;Rate}_{SALUS}\times {Days\;of\;regrowth}_{i}$$ (3)

where *Growth Rate*_*SALUS*_ is the pasture growth rate (kg DM ha^-1^d^-1^) estimated by SALUS and *Days of regrowth* is the number of days between the last grazing event (input by the user) in each paddock (*i* = 1-*n*) and the last UAV flight.

Additionally, the pre-grazing pasture biomass and days to achieve a given pre-defined pasture biomass target (input by user) were calculated as follows:

$${Pre\;grazing}_{i}={UAV}_{i}+{Growth\;Rate}_{SALUS}\times {Days\;in\;rotation}_{i}$$ (4)

$${Days\;to\;target}_{i}=\left(Target-{UAV}_{i}\right)/{Growth\;Rate}_{SALUS}$$ (5)

where *Pre-grazing*_*i*_ is the predicted pasture biomass (kg DM ha^-1^) at a given user-defined rotation length (i.e. days of resting or grazing interval) and *Days to target*_*i*_ is the number of days to achieve a given user-defined pre-grazing pasture biomass (Target, kg DM ha^-1^) for each consecutive paddock (*i* = 1-*n*).

In practice, *Pre-grazing* and *Days to target* provide useful information and alternatives for different grazing management schemes. The former is used when the grazing schedule is based on fixed-length rotation and the latter is used when it is based on the maintenance of a fixed pre-grazing and post-grazing pasture biomass (i.e. predefined grazing wedge). The present UAV-based pasture modeling approach calculates both alternatives.

### Modeling morphology and digestibility of pasture using MDP model

The MDP model incorporated the predicted pasture biomass data to simulate key nutritive value parameters. The MDP model is described in detail by [10]. Briefly, it involves the use of daily temperature and post-grazing (residual) pasture data to drive a leaf morphogenetic model that predicts plant morphology traits (i.e. leaf number and length) and digestibility of pasture. The distinctive feature of the MDP model is that it takes into account the effect of grazing (severity and frequency) on leaf morphogenesis (appearance, growth and senescence) and digestibility of pasture fiber [19]. The model closely predicts variation of pasture dry matter digestibility (DMD, %) during regrowth by simulating the dynamics of its components, neutral detergent fiber (NDF, %), and NDF digestibility (NDFD, %), all related to modelled changes in the leaf stage and leaf length of plants.

Parameters for key leaf morphogenetic and nutritive value variables (S2 Table) were implemented in the MDP model to represent both Fescue and Ryegrass pasture and by using both tall fescue and perennial ryegrass as dominant plant species, respectively. Parameters for plant morphology and nutritive value of Fescue were set as default and were based on a robust dataset that included several tall fescue regrowth experiments [10]. For the case of Ryegrass, the faster leaf turnover of perennial ryegrass compared to tall fescue [20] was properly represented by reducing the leaf lifespan from 630 to 330 degree days (°Cd) [21]. Likewise, both Ryegrass leaf appearance and elongation rate parameters were increased two-fold in comparison to Fescue. Finally, in order to reflect the lower fiber content and higher digestibility for Ryegrass pasture compared to Fescue pasture [22–24], both the minimum NDF and maximum NDFD parameters were both reduced and increased by 5 and 20 percentage units, respectively.

### Experiment 1: UAV calibration and models validations (plot scale)

The first experiment (Exp. 1) was carried out during spring (May 6 to June 9) and summer (June 21 to August 15) of 2016 using an enclosed pasture area of the KBS dairy farm. The experiment included both measurements of pasture and UAV flyovers, both used for: i) calibration of the UAV pasture measurement technique, and ii) both testing of the SALUS and the MDP model. Briefly, typical on-farm variation in pasture cover was generated by using three residual (i.e. initial state of regrowth) pasture treatments, a low (3 cm), medium (6 cm), and high (12 cm) pasture stubble, respectively. The three pasture residual sward height treatments were randomly applied and assigned to main plots following a split-plot design replicated across three blocks. Main plots (n = 18, 2 species x 3 residual treatment x 3 blocks) were further subdivided into six subplots, which were randomly assigned to six consecutive harvest dates. Pasture growth across the six consecutive harvests was then performed every 7–10 days. Before each harvest event, the canopy leaf area index was measured using a LAI-2000 plant canopy analyzer (LI-COR Inc, Lincoln, NE, USA). Concurrently, the herbage mass accumulation at each harvest event was determined by clipping a randomly placed 0.25 m^2^ quadrat of pasture to ground level. The material harvested was immediately bagged and oven-dried at 60°C for 48 h to estimate the accumulated DM biomass (kg DM ha^-1^).

In tandem with harvesting events, intact tillers of tall fescue or perennial ryegrass were removed from two of the blocks of Fescue and Ryegrass, respectively. Seven tiller samples (harvest II, III, IV, V for spring regrowth and harvest II, IV, VI for summer regrowth) were collected per residual sward height treatment. At each of the 84 sampling events (3 treatments x 2 pastures x 7 harvest x 2 blocks), selected tillers (~1000 per block) were obtained from randomly placed quadrats (0.25 m^2^). Plants were cut at the tiller base and pooled to form one sample per replicate. The plant material was immediately stored at -20°C until further processing. In the laboratory, the material was thawed and plant material from ~300 tillers was randomly selected. Leaves of the selected tillers were separated into leaf blade and leaf sheath components both for leaf morphological (leaf length and leaf number per tiller) determination and nutritive value analysis. Thereafter, leaf blades were dried to constant weight and ground to pass 1 mm screen for determination of NDF with an ANKOM 200 fiber analyzer (ANKOM Techn., New York, USA), and 24 h in vitro digestibility using a Daisy^II^ apparatus (ANKOM Techn., New York, USA). The 24 h NDFD [(NDFincubated–NDFresidual) / NDFincubated] was estimated [25]. Finally, the 24 h apparent DMD was calculated by subtracting the metabolic factor 119 mg kg^-1^ [26] from the 24 h true DMD [1- (NDFresidual/ weight of incubated DM)], [25].

#### UAV calibration

The UAV-derived NDVI was calibrated against the pasture biomass harvested from the 18 georeferenced plots during the first four harvests events of summer regrowth. The biomass accumulated (kg DM ha^-1^) per plot was related to the mean NDVI value determined by UAV remote sensing immediately before each harvest. The NDVI data comprised ~1200 pixel measurements across each 8 m^2^ plot (6 cm of resolution). The average NDVI value of all pixel points per plot (excluding the quadrat area sampled for previous harvests) was calculated and then regressed (Eq 2) against the actual pasture biomass that was previously determined by clipping.

#### Models evaluation

The SALUS and MDP models were evaluated by the comparison between simulated and observed data from Exp. 1. Evaluation analysis of SALUS model performance in Exp. 1 was recently conducted in [15] (S1 Table). For MDP evaluation the ability of the model to predict plant morphology (length and number of leaves per tiller) and digestibility of pasture was tested in this study.

The experimental Fescue and Ryegrass regrowths for spring and summer season were simulated by using the same weather and residual pasture height of experiment 1. Temperature (°C) was used as daily input data and residual pasture height was specifically adjusted for each of the three treatments, 3, 6 and 12 cm for the low, medium and high residual pasture height treatment, respectively. Following the statistical analysis proposed by [27], linear regression between observed and simulated data was analyzed for intercepts and slope values of 0 and 1, respectively (accuracy), as well as for the coefficient of regression (R^2^, precision). Simulated and observed data were compared by deviations, root mean square error (RMSE) and relative prediction error (RE), as follows:

$${Deviation}_{i}={Simulated}_{i}-{Observed}_{i}$$ (6)

$$RMSE=\sqrt{\frac{\sum_{i=1}^{n}{\left({Deviation}_{i}\right)}^{2}}{n}}$$ (7)

$$RE=\frac{RMSE}{\overset{-}{X}}\times 100$$ (8)

where *Simulated*_*i*_ is simulated value_*i*_, *Observed*_*i*_ is observed value_*i*_, *n* is the total number of observations, $\overset{-}{X}$ is the mean of observed values over *n*, and *i* is the *i-*th observation. Finally, the extent of agreement between simulated and observed values was tested by the concordance correlation coefficient (CCC) which is a simultaneous measure of accuracy and precision with an ideal fit indicated by a value of 1, and the bias correction factor (Cb) which indicates degree of bias from the y = x line with a value of 1 indicating no bias.

### Experiment 2 (farm scale)

A second on-farm grazing experiment (Exp. 2) was carried out for a 4-week period (24 days) during summer of 2016. Protocols for animal handling and husbandry have been previously revised, approved and applied according to the Michigan State University’s Institutional Animal Care and Use Committee (IACUC) office, under project application number 02/14. The milking herd comprised 66 US Holstein-Friesian cows. A total of sixteen 1-ha paddocks (1 ha each) were used for this experiment and were divided into 8 Fescue and 8 Ryegrass 1-ha paddocks. Paddocks were connected via two-way laneways to a centralized free-stall robotic milking barn for voluntary milking. The daily diet of cows comprised primarily grazed pasture plus an addition of pellet concentrate and free choice of a molasses-based mineral and vitamin supplement. The pellet concentrate was fed during milking at a rate of 1 kg DM of pellet every 6 kg of milk, and up to a maximum of 7 kg DM d^-1^. Cows had 24 h access to pasture through the use of selective sorting gates located at the south end of the barn. Each day, cows received a daily allowance of ~25 kg DM cow^-1^ of fresh pasture in two breaks of ~12.5 kg DM cow^-1^ that were offered on alternating locations of the farm (north and south); this 2-way grazing system (i.e., A-B grazing schedule) was decided for improved voluntary cow traffic and milking of cows [28]. Pasture allocations were made available from 1000 to 2200 h and from 2200 to 1000 h, respectively. Additionally, the criteria to adjust the size of pasture allocations also included the following pasture management rules, the use of a pre-grazing pasture biomass target of 2700 kg DM ha^-1^, the use of a post-grazing pasture biomass target of 1300 kg DM ha^-1^, and the maintenance of an average pasture cover of no less than 1800 kg ha-1 across the entire grazing platform. The size of the daily allocation of pasture varied in relation to animal consumption and pasture growth rate, and resulting on average in 1 to 3 grazing days per 1-ha paddock.

Weekly pasture biomass of each paddock was systematically monitored alongside transects by using a calibrated Rapid Pasture Meter (C-Dax; Agricultural Solutions, Ltd, Palmerston North, New Zealand). The C-Dax meter consists in a trailer-like attachment that is towed by an all-terrain vehicle. The equipment uses optical sensors to measure plant height at very high frequency (200 MHz), providing geospatial pasture data every ~2.7 m. The original average pasture height (x, mm) recorded by the C-Dax was converted into average pasture biomass (y, kg DM ha^-1^) according to two previous formulas, one for Fescue (y = 15x-150, R^2^ = 0.82, n = 32) and one for Ryegrass (y = 17x-57, R^2^ = 0.75, n = 72). By using a GPS device (± 1 m) mounted to the equipment, the C-Dax can provided accurate data both of pasture mass and location [29]. The geospatial pasture data was downloaded and transferred into a GIS database for mapping and spatial analysis.

In addition to C-Dax measurements, pasture mass in each paddock was estimated by the sward ruler method by using 23±2 sward height readings alongside a parallel linear transect to the C-Dax track. Sward surface height was measured from ground level using a first-contact technique [30] with a Hill Farming Research Organization sward stick [30, 31]. A unique pre-defined equation for both pastures (y = -515 + 123x; R^2^ = 0.86; n = 90) was developed on site to convert average ruler sward height (x, cm) into average herbage mass (y, kg DM ha^-1^).

#### On-farm evaluation of the UAV monitoring method

**Fig 2:**
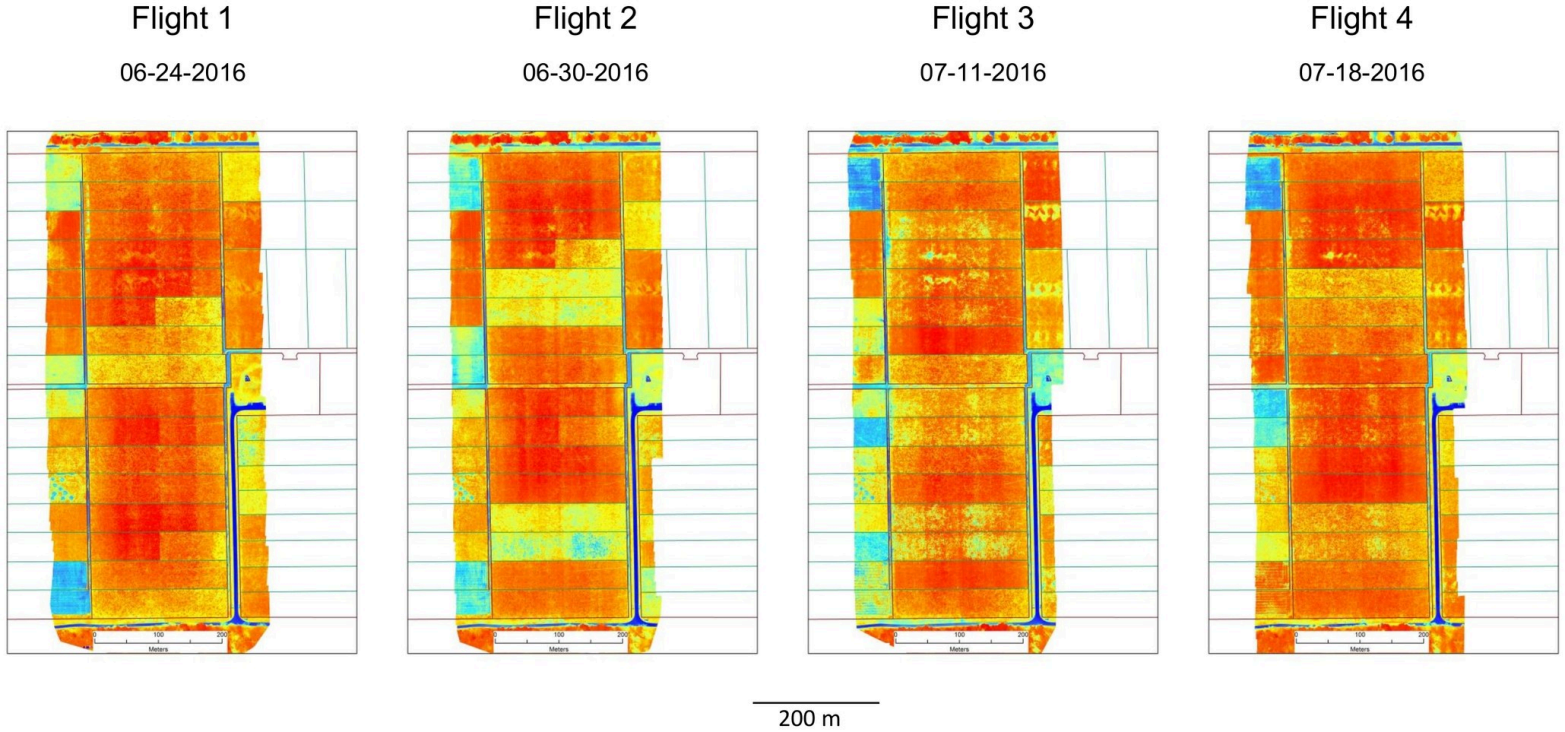
UAV-derived NDVI maps of grazed pasture of the KBS dairy farm (area of 15.6 ha) collected on four consecutive dates. Maps exhibit the spatial variability of high-resolution measurements (6 cm pixels) of pasture NDVI (-1: blue to 1: red color) in two blocks of eight 1-ha paddocks (200 x 50m) used for rotational grazing with lactating dairy cows.

Four UAV flyovers were performed in June 24 and 30, and July 11 and 18, 2016 to collect multispectral data and to develop NDVI raster maps (Fig 2). Flights were conducted ~8:30 am on same pasture monitoring days and immediately before field-based methods, C-dax and ruler. Following each flight, the paddock average biomass was estimated from the paddock-average NDVI according to the calibration equation (Eq 2) developed previously during the UAV calibration phase. All UAV flights were conducted at 100 m elevation and by using the same west-east transect flight pattern across fields. On average, four flight transects were needed to cover the entire experimental area with a 75% horizontal overlap between transects. The NDVI-derived biomass for each paddock was compared against the average biomass determined by the two field-based methods, C-Dax and ruler. Technical information for the three monitoring methods is presented in Table 1.

**Table 1:** Characteristics of three methods of pasture monitoring used at the KBS dairy farm. Technical data of Ruler, C-Dax and UAV based methods of pasture monitoring used during four consecutive weekly dates at the KBS dairy farm. Values in table represent reference data for a 1-ha paddock (200 x 50 m).

Technical data	Ruler	C-Dax	UAV
N° observations	23	70	2,000,000
Frequency (m)	10	2.7	0.06
Time to cover (sec)	300	45	300
Speed (m/sec)	Walking	5	12
Measurement height (m)	-	0.02	100
Calibrated equations	1	2	1

Comparison of methods was conducted as follows. First, vector maps of C-Dax sampling transects were imported into the ArcGis database and approximately 70±6 spatially explicit measurement points were identified for each 1-ha paddock transect and monitoring date. Second, the average NDVI-derived biomass enclosed within a 1 m diameter around C-Dax sampling points was intersected and calculated. Finally, the mean NDVI-derived biomass for each paddock transect and sampling date was calculated and compared to that of the C-Dax and ruler methods by calculation of the mean deviation and RMSE, as follows:

$${Deviation}_{i}={UAV}_{i}-(\frac{{CDax}_{i}+{Ruler}_{i}}{2})$$ (9)

$$RMSE=\sqrt{\frac{\sum_{i=1}^{n}{\left({Deviation}_{i}\right)}^{2}}{n}}$$ (10)

where *UAV*_*i*_, *CDax*_*i*_ and *Ruler*_*i*_ are the pasture biomass estimated by the UAV, C-Dax and ruler, respectively, for each consecutive paddock and date (*i* = 1-*n*). Additionally, the regression line between UAV-derived biomass (*x* axis) and mean biomass of C-Dax and ruler (*y* axis) was analyzed for intercept and slope values of 0 and 1, respectively (accuracy), as well as for coefficient of regression (R^2^, precision).

#### UAV-modeling system outputs

Once the three main components (UAV, SALUS and MDP models) were evaluated, main outputs of UAV-modeling data were summarized and presented for the four flights. The area coverage for this synthesis included the 16 ha of pasture of the KBS dairy farm covered during the four UAV flyovers. Weather data (rainfall, solar radiation and temperature) recorded at a meteorological station located at the experimental site were used as daily input for the SALUS and MDP models.

## Results

### UAV system

**Fig 3:**
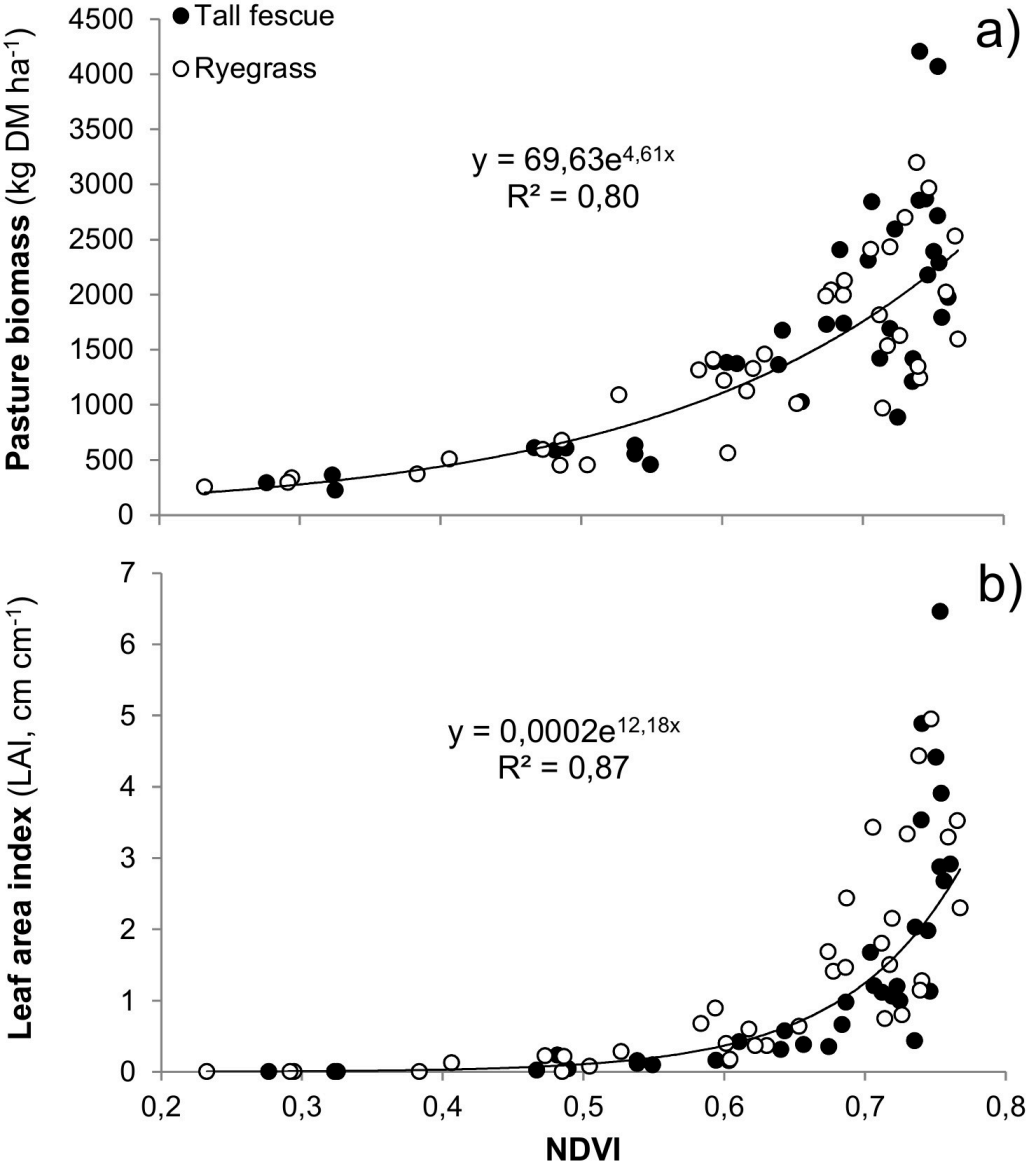
UAV calibration. Relationship among UAV-remotely sensed NDVI and pasture biomass (a) and leaf area index (b) in georeferenced plots of tall fescue- (black) and ryegrass-based (white) pasture plots (n = 72) in Exp. 1.

During Exp. 1, pasture biomass ranged from 226 to 4208 kg DM ha^-1^ for Fescue and from 255 to 3200 kg DM ha^-1^ for Ryegrass, respectively. Likewise, the NDVI for same plots was 0.28 to 0.76 for Fescue and 0.23 to 0.77 for Ryegrass, respectively. Similarly, the measured LAI ranged from 0.01 to 6.5 for Fescue and 0.01 to 5.0 for Ryegrass, respectively. The NDVI derived from multispectral imagery collected by the UAV system (n = 72 plots) was strongly related (p = 0.001) both to the measured pasture biomass (R^2^ = 0.80) and LAI (R^2^ = 0.87; Fig 3). When NDVI was regressed against biomass, the coefficients for non-linear regressions of Fescue and Ryegrass did not differ (p = 0.38), supporting a common NDVI-based herbage mass predicting equation for both species (Fig 3a).

**Fig 4:**
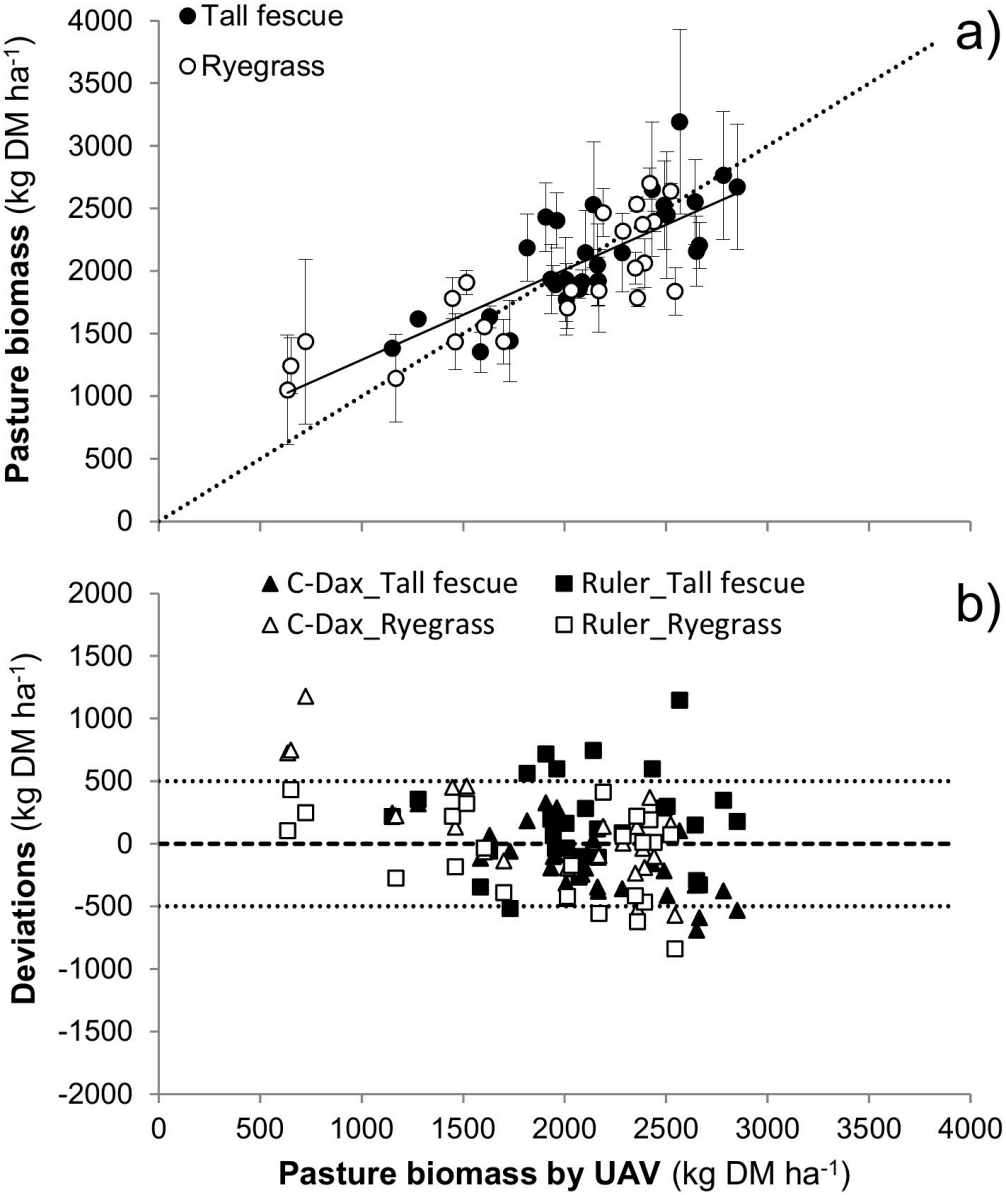
Evaluation of UAV method. Evaluation of the UAV method for estimation of biomass of tall fescue- (solid symbols) and ryegrass-dominated (open symbols) pasture for four sampling dates conducted during Exp. 2. (a) Graphical representation of goodness of fit of pasture biomass estimated by the UAV compared to mean reference values estimated by the two control methods, ruler and C-Dax. (b) Deviation of pasture biomass estimations by the UAV method compared to the ruler (■□) and C-Dax (▲Δ) methods.

Results of pasture biomass estimations of Exp. 2, using the UAV, C-Dax and ruler methods are presented in Table 2. Estimations of pasture biomass by the UAV (1971±350 kg DM ha^-1^) were similar (p > 0.05) to estimations by the C-Dax (2073±636 kg DM ha^-1^) and ruler (2017±530 kg DM ha^-1^) methods, respectively. Consequently because there was no difference (p > 0.05) between control methods, data from the C-Dax and ruler methods were pooled and used for comparison of the UAV performance. Fig 4a shows that the UAV method provided similar estimations that fell within the range of the one standard deviation by the C-Dax and ruler methods. Further, in almost all cases (85% of observations), the difference in pasture biomass estimations among methods fell within the range of ± 500 kg DM ha^-1^.

**Table 2:** Summary of statistics and comparison of the UAV-based method for estimation of herbage mass of tall fescue- and ryegrass-based pastures rotationally grazed by lactating dairy cows. Comparisons were performed for each 1-ha paddock during the four pastures’ monitoring dates in Exp. 2.

	Biomass estimation (kg DM ha^-1^)			Comparison vs UAV					
	Mean	SD	n	Bias	t-test	RMSE	RE	R^2^	r
UAV	2017	530	52	-	-	-	-	-	-
C-Dax	1971	350	52	-46	0.37	363	18	0.53	0.73
Ruler	2073	636	52	56	0.30	386	19	0.63	0.80
Mean	2022	472	52	5	0.90	313	15	0.65	0.81

### SALUS model

The results for the evaluation of the SALUS for estimation of pasture growth were recently reported by [15] and it is summarized in S1 Table. The SALUS model adequately simulated the herbage mass (RE = 19%, RMSE = 509 kg DM ha^-1^, CCC = 0.94) of spring and summer regrowth, with a slightly better estimation for Ryegrass (RE = 14%, RMSE = 390 kg DM ha^-1^, CCC = 0.93) compared to Fescue (RE = 22%, RMSE = 590 kg DM ha^-1^, CCC = 0.92). Overall, the ability of SALUS to effectively simulate the effect of different residual sward height treatments was reflected by high values for the following model fit statistics: R^2^ = 0.89, r = 0.94 and Cb = 0.99, respectively.

### MDP model

**Fig 5:**
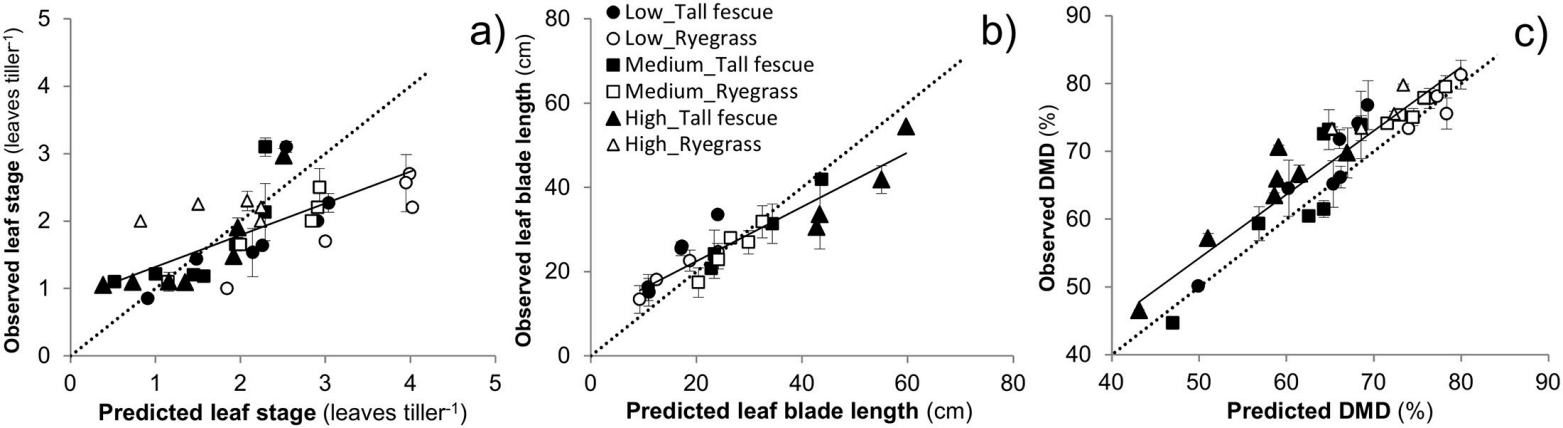
Evaluation of the MDP model. Predicted vs. observed leaf stage (a), leaf blade length (b) and digestibility of dry matter (c) of spring and summer regrowth of tall fescue- (solid symbols) and ryegrass-based (open symbols) pasture managed under three residual sward height treatments (low, ●○; medium, ■□; high, ▲Δ) in Exp.1.

Reduction of residual sward height in Exp. 1 shortened (p < 0.001) the length of consecutively produced leaves in spring and summer, both improving the NDFD and DMD of Fescue (p < 0.02) and Ryegrass (p < 0.04) pasture. Overall, the MDP model adequately predicted leaf length and leaf stage, and the NDF, NDFD and DMD of pasture (Table 3). The length of leaves (RE = 23%, RMSE = 6.2 cm, R^2^ = 0.92) was predicted with greater accuracy compared to predictions of leaf stage (RE = 39%, RMSE = 0.7 leaves, R^2^ = 0.70), and the leaf stage was predicted with greater accuracy for Fescue pasture (RE = 29%, RMSE = 0.5 leaves, R^2^ = 0.60) compared to Ryegrass (RE = 46%, RMSE = 0.9 leaves, R^2^ = 0.33) pasture (Fig 5a). There was a good agreement between the observed and simulated DMD (Fig 5c), and both the DMD and NDFD (RE = 8%, RMSE = 5%, R^2^ = 0.74) were simulated with better accuracy compared to simulations of NDF content (RE = 16%, RMSE = 9%, R^2^ = 0.77).

**Table 3:** Summary of statistics for testing of the MDP model for prediction of leaf morphogenetic traits and forage nutritive value of tall fescue- and ryegrass-based pasture. Mean, standard deviation (SD), mean bias, root mean square error (RMSE), relative error (RE), coefficient of the regression (R^2^) and Pearson correlation coefficient (r) for leaf length (cm), leaf stage (leaves per tiller), neutral detergent fiber content (NDF, %), and digestibility of dry matter (DMD, %) and NDF (NDFD, %), both of summer and spring regrowth managed with different residual sward heights in Exp. 1.

	Mean		SD		Bias	RMSE	RE	R^2^	r	CCC	Cb	n
	Actual	Simulated	Actual	Simulated								
Leaf length												
All	27.3	27.4	9.8	14.1	-0.1	6.2	23	0.85	0.92	0.62	0.67	22
Fescue	30.4	31.2	11.1	16.4	-0.8	7.6	25	0.82	0.91	0.68	0.75	13
Ryegrass	22.9	22.0	5.8	7.6	0.9	3.1	13	0.86	0.93	0.82	0.89	9
Leaf stage												
All	1.8	2.1	0.6	0.9	-0.2	0.7	39	0.49	0.70	0.65	0.93	36
Fescue	1.7	1.7	0.7	0.8	0.0	0.5	29	0.60	0.78	0.67	0.86	21
Ryegrass	2.0	2.5	0.5	1.0	-0.5	0.9	46	0.33	0.58	0.45	0.79	15
NDF												
All	51.5	53.0	4.8	3.2	-1.4	3.4	7	0.59	0.77	0.71	0.93	36
Fescue	56.8	50.3	12.7	11.2	6.5	8.8	16	0.77	0.88	0.57	0.71	21
Ryegrass	48.8	50.2	2.8	2.5	-1.4	2.8	6	0.31	0.55	0.55	0.99	15
NDFD												
All	64.8	59.3	13.8	14.4	5.5	7.8	12	0.85	0.92	0.92	1.0	36
Fescue	53.5	54.9	5.1	2.1	-1.4	3.8	7	0.66	0.81	0.87	0.99	21
Ryegrass	75.9	71.8	4.5	7.0	4.1	6.0	8	0.58	0.76	0.69	0.91	15
DMD												
All	69.4	66.1	9.2	9.0	3.3	4.8	7	0.86	0.93	0.93	1.0	36
Fescue	64.5	60.6	9.0	7.4	3.9	5.4	9	0.82	0.91	0.89	0.98	21
Ryegrass	76.2	73.8	2.6	4.1	2.4	3.6	5	0.56	0.75	0.68	0.91	15

### UAV-modeling integrated system

**Fig 6:**
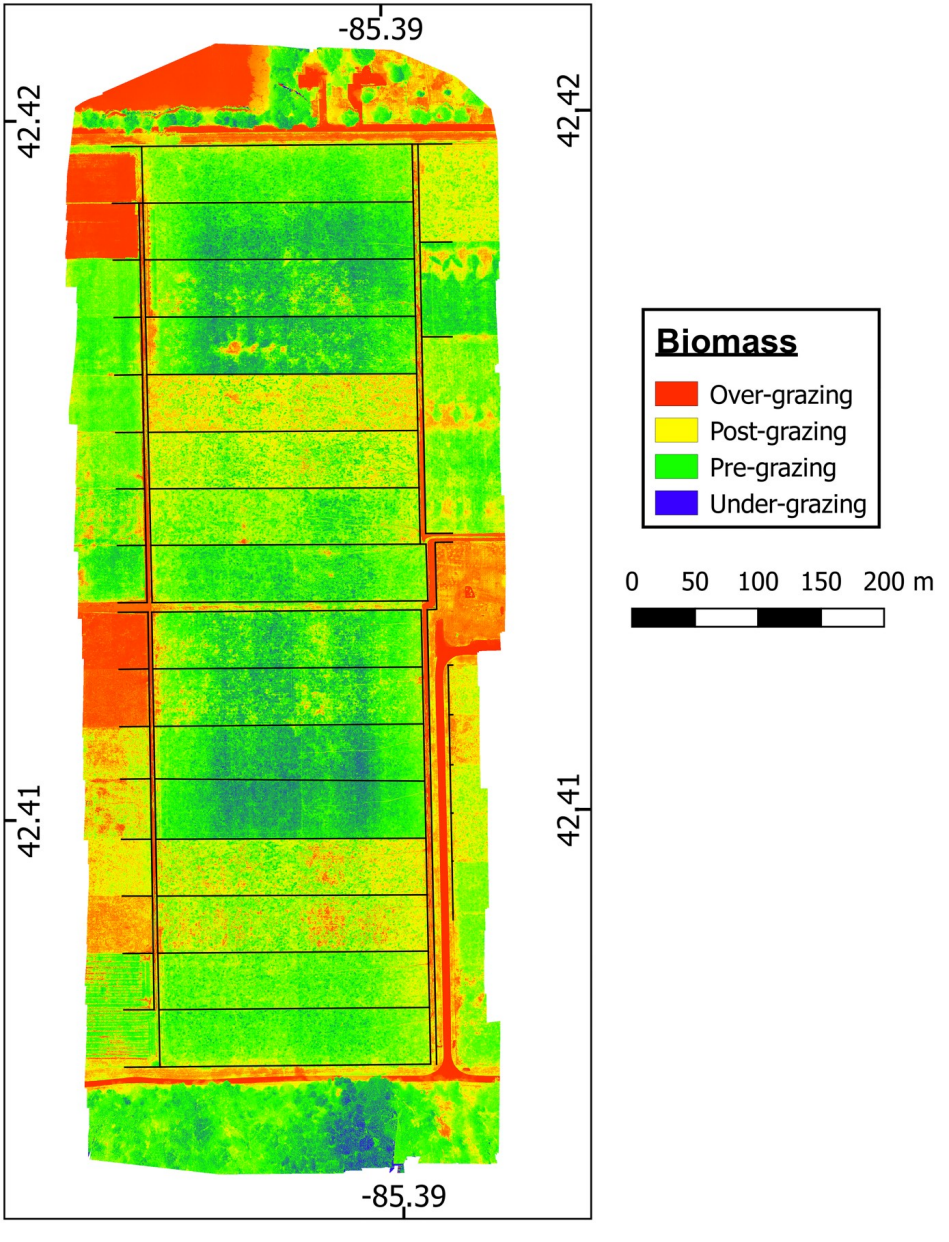
Example (Flight 4) of output map of estimated pasture biomass for a platform of 15.6 ha rotationally grazed with lactating cows in Exp. 2. The map shows high-resolution (6 cm) spatial variability of pasture cover with colors denoting <500 kg DM ha^-1^ (red), 500–1700 kg DM ha^-1^ (yellow), 1700–2800 kg DM ha^-1^ (green) and > 2800 kg DM ha^-1^ (blue).

**Fig 7:**
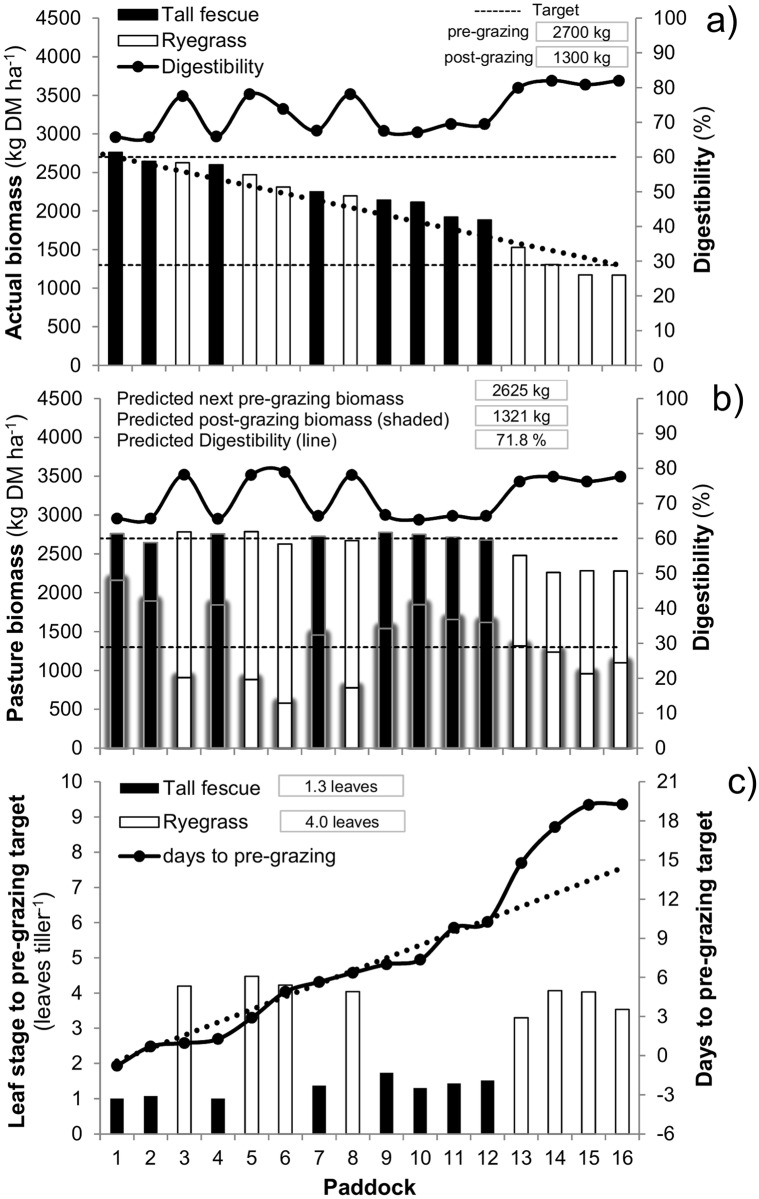
Main outputs of the UAV-modeling system for Flight 4 (corresponding to Fig 6). (a) Actual pasture cover and model estimation of digestibility (DMD, %) for each paddock in rotation. (b) Model estimations showing the predicted post-grazing (shaded bars) and both the predicted next pre-grazing biomass, digestibility (DMD, %) for each paddock in rotation. (c) Model estimations of leaf stage (leaves per tiller) and number of resting days needed to achieve the predefined pre-grazing target of 2700 kg DM ha^-1^ for all paddocks in rotation. The dotted line indicates the ideal resting days in relation to the length of rotation.

**Fig 8:**
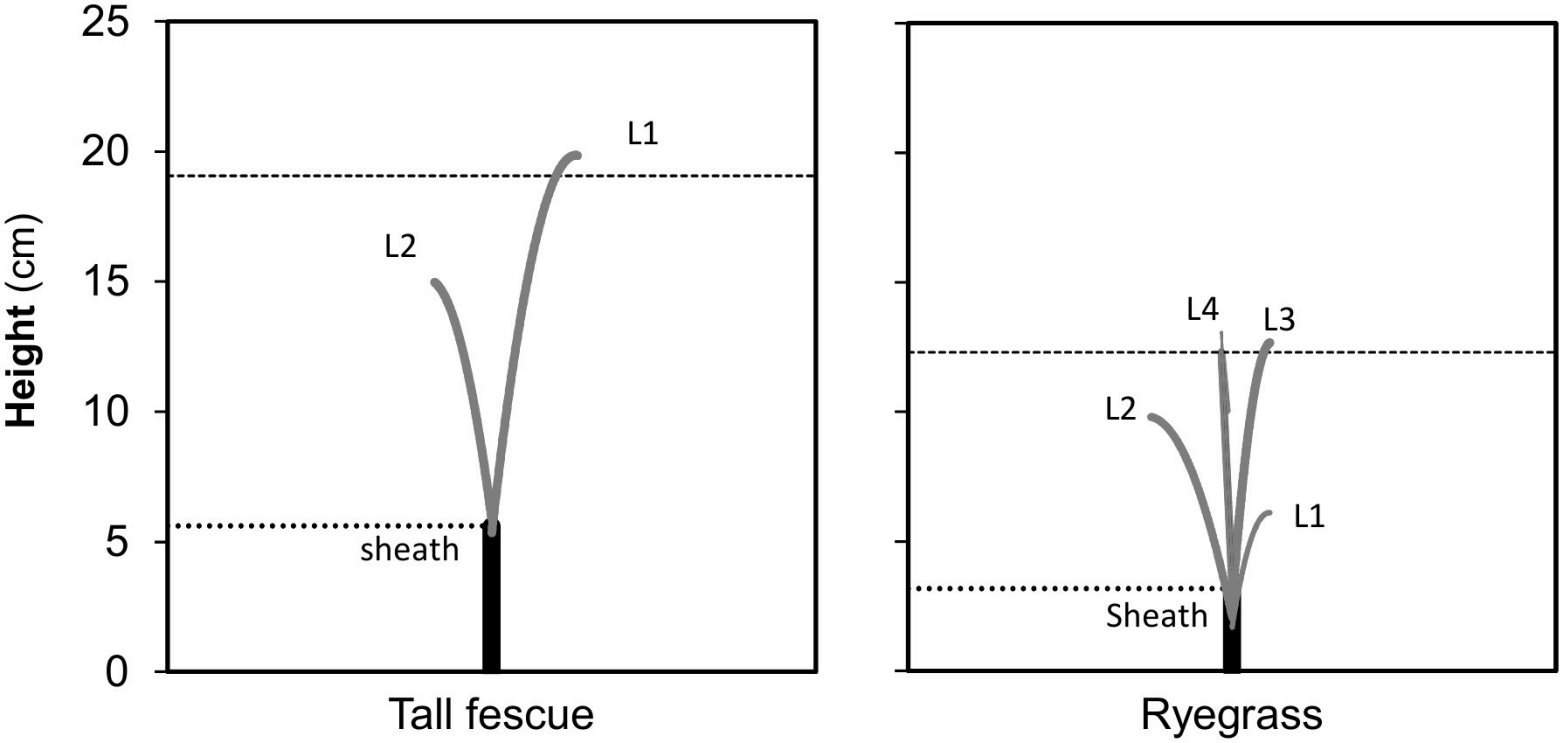
Schematic representation of the leaf morphology and leaf stage of tillers simulated by MDP model. Figures correspond to the average 1.3- and 4.0-leaf stage of plants recommended for tall fescue and ryegrass in order to achieve the predefined pre-grazing target of 2700 kg DM ha^-1^.

The results for on-farm estimation of pasture biomass, morphology and digestibility for paddocks of the rotational grazing system of the KBS dairy farm are reported in Figs 6–8. The spatial variability of estimated biomass is shown in Fig 6 and the estimated mean biomass and forage digestibility for each paddock is shown in Fig 7a. The coupled UAV modeling system predicted both the quantity and nutritive value of forage for each paddock based on the expected rotation and resting time that was needed to achieve a set pre-grazing target of 2700 kg DM ha^-1^. This predicted information is shown in Fig 7b together with the predicted residual pasture biomass values. Figs 7c and 8 show the actual leaf stage for each paddock as a graph and as a scheme, respectively. Fig 7c also shows the calculated resting days required to achieve the predefined pre-grazing biomass target of 2700 kg DM ha^-1^.

## Discussion

The aim of this work was to develop and test an integrated systems approach to monitor, assess and plan grazing management on farms (Fig 1). This novel system is based on the integration of remotely collected data by UAV-mounted sensors and process-based models. Overall, the results reported in this paper provide sufficient evidence both to support the feasibility of the proposed approach and its validation by using data both from plot and field scale experiments. The results also suggest potential for scalability of the present UAV- modeling approach to other grass species, soils and weather conditions, but the testing and validation of this hypothesis warrants further carefully conducted investigations.

### Pasture monitoring with UAV

Recent studies have shown great potential for UAV applications in forestry and agriculture (e.g. [3, 32–34]) but this modern technology has not yet been used to assess pasture growth in livestock grazed systems. In the present study, the strong relationship shown (R^2^ = 0.80) between NDVI values derived by UAV-mounted sensors and the actual biomass measured on geo-referenced plots (Fig 3) supported the use of UAV-derived NDVI values to estimate pasture biomass.

Previous NDVI values using ground-based sensors in tall fescue have been reported to range from 0.53 to 0.78 [35]. Similarly, UAV-derived NDVI registered from Fescue and Ryegrass plots in Exp. 1 ranged from 0.23 to 0.78. As observed in other species [36–38], the curvilinear relationship observed between the NDVI and biomass (Fig 3a) shows that this vegetation index increased with pasture growth up to a maximum NDVI value of ~0.8. This typical saturation of NDVI occurred when the LAI value of pasture was sufficiently high (Fig 3b) to minimize reflectance of red light associated to high soil cover [39]. In this sense, [40] observed that the NDVI had a reduced biomass prediction capability with values of LAI greater than ~3. Consistent to this finding, our results demonstrate that the present UAV technology can be used to monitor well-managed grazed pastures that usually do not exceed ~3000 kg DM ha^-1^ of pre-grazing cover or a LAI of 3; greater biomass or LAI will certainly lead to more poor pasture biomass prediction.

The scalability of the UAV for prediction of pasture biomass at paddock and farm scales was confirmed during Exp. 2. Pasture biomass at paddock scale determined from UAV-derived NDVI values was similar to concurrent biomass estimations by two more commonly used pasture monitoring transect methods, the ruler and the C-Dax pasture meter (Table 2, Fig 4a). Overall, the mean difference between methods was less than 400 kg DM ha^-1^. Moreover, only 15% of observations from the entire dataset had an estimation difference greater than ± 500 kg DM ha^-1^ (Fig 4b), and almost all of those were comparisons of post-grazing herbage mass estimations (i.e. low pasture biomass) associated to low soil cover or greater quantity of senescent vegetation or litter. Consequently, these underestimations (Fig 4a) were usually due to the fact that UAV-mounted sensors discriminated dead from green plant material by increasing red light reflectance and by decreasing NDVI values (and pasture cover estimations), whereas estimations by the other two more traditional pasture monitoring methods did not discriminate between dead and green plant tissue. The present results therefore suggest that the UAV system could be a more adequate method to assess more nutritious green plant components, which indeed is the fraction of pasture that is more likely to be consumed in a rotational grazing system.

The high measurement frequency (6 cm) combined to a greater spatial extent and resolution of UAV data also allowed for more representative estimations of pasture biomass compared to the conventional control methods (Table 1). Further, this high measurement frequency may solve possible issues related to a weak UAV calibrations (i.e. < R^2^), especially in situations with large spatial variability of pasture associated to uneven animal grazing. This is perhaps one of the most remarkable applications of the UAV method, in particular if the extent and sampling intensity of the UAV method is compared to the more limited capabilities of more common pasture monitoring methods used by dairy farmers [8]. Finally, spatial patterns of pasture from UAV-derived NDVI values (Figs 2 and 7) are of a much finer scale than the mean of pasture biomass predicted by conventional methods on a single linear transects per paddock. This offers the potential to quantify pasture biomass with high spatial resolution, map large areas (Fig 6) or gain relevant information to understand and manage the spatial variability of pasture associated to uneven grazing behavior (i.e. patch grazing), landscape heterogeneity, or soil properties [41]. These findings further suggest that the average pasture cover per paddock as estimated by UAV-mounted sensors would represent the most meaningful pasture biomass data for accurate forage allocation to animals in pasture-based livestock systems.

### Prediction of pasture growth

A proactive approach to grazing management must consider both the actual pasture cover to manage forage allocations, and the change in pasture growth trends to either avoid over- or under-grazing in a grazing rotation (e.g. grazing paddocks either too soon or too late). Reasonably, predictions of pasture growth need to consider both the prevailing meteorological conditions and the actual soil properties. Therefore, a unique feature of the present approach is the integration of high-resolution UAV data and process-based models to predict pasture growth based on actual soil, plant and weather components (Fig 1). A similar approach combining both field measurements and information obtained by a simulation model has been used by [42] to help farmers to reduce the frequency of pasture monitoring.

In the present study, SALUS was used as the model to estimate pasture growth and cover up to ~10 days after the last UAV flyover. This short-term simulation offered both, the opportunity to estimate quickly and accurately post-grazing residuals left by animals (shaded bars in Fig 7b) and to predict the number of days that would be needed to achieve the desirable pasture pre-grazing target, usually predefined by farmers by the setting of a targeted grazing wedge (line in Fig 7c). Eventually, our approach also predicted the next pre-grazing biomass at a given length of rotation (bars in Fig 7b), which is a useful piece of information for rotational systems that are grazed at fixed day intervals. Finally, prediction of leaf stage tiller data (Figs 7c and 8) could be used as companion information to apply plant-related indicators to further guide grazing management [43]. Thus, multiple sources of model information can be integrated and used to drive informed decisions for grazing management, including adjustments of pasture allocations, changes in grazing rotations, decisions of pasture harvest, or use of feed supplementation. For example, as shown by Fig 7b the actual biomass across paddock (Fig 7a) might allow reaching the desired pre-grazing target of 2700 kg DM ha^-1^ (future situation) in most of the paddocks, except for the last three paddocks in the rotation (with bars below the targeted wedge line). Consequently, Fig 7c predicts that the next grazing rotation must be extended by ~3 days, otherwise an expected decrease of ~400 kg DM ha^-1^ of pre-grazing pasture cover over the last three paddocks (Fig 7b) will imply that additional supplementary feed would be needed to offset pasture deficits and to avoid over-grazing or decline in pasture intake. Finally, it is important to acknowledge that the present UAV-modeling approach to predict and guide management of pasture growth was based on a multistep process that has potential to aggregate errors across estimations. Therefore, further testing of the present UAV-modeling approach, including scalability to other pasture systems would be needed for more reliable testing and application on farms.

### Estimation of pasture digestibility

The nutritive value of pasture is a dynamic plant-related trait that can significantly affect animal performance [44]. For most farmers the more commonly used trait to assess pasture nutritive value is digestibility, which is an accurate reference for the actual feed value of most forages. Importantly, the present testing of the MDP model to predict digestibility of Fescue and Ryegrass pasture managed with different defoliation heights (Table 3 and Fig 5) supports the ability to proactively provide information of pasture nutritive value to guide grazing management (Fig 7b and 7c).

Criteria to determine timing of grazing quite often is based on a fixed number of days, a targeted herbage mass or a given number of leaves per tiller (i.e. leaf stage). However, the present UAV-model approach provided more precise information to decide grazing management both based on estimations of cover and forage digestibility. This is perhaps the most noteworthy feature of the present UAV-modeling system since changes in nutritive value can significantly affect the voluntary intake of pasture [44] and animal performance, in particular when lower pasture nutritive value related to high pasture cover (> 3500 kg DM ha^-1^) is related to the use of very high pre-grazing targets [5] and/or very long grazing rotations in relation to the onset of leaf senescence [19]. For example, Fig 7a and 7b indicate that during the present grazing experiment (Exp. 2) dairy cows were provided consistently high-quality forage associated to a fast grazing rotation (< 14 days). Consequently, this rotation speed resulted in grazing intervals that were shorter (< 325 °Cd) than the leaf lifespan of the grass species [20], thereby avoiding rapid increase in NDF and decrease in NDFD associated with increasing leaf senescence [19]. Finally, it is important to acknowledge that a greater temporal and spatial variation in pasture nutritive value could be expected in commercial farms where grazing plans are not carefully monitored and controlled as in the present grazing experiment, and therefore scalability of the present UAV- and model-based approach for use in other farms and systems warrants further carefully conducted investigations [45].

## Conclusion

The present UAV-modeling approach integrated pasture NDVI data remotely collected by UAV-mounted sensors and process-based models for prediction of pasture growth, morphogenesis, and forage nutritive value. Both, results from plot and filed scale experiments provide sufficient evidence to support feasibility for the UAV-modeling approach and potential for broad scalability to other grass species, soil types and weather conditions, but the testing and validation of this hypothesis warrants further carefully conducted investigations.

Importantly, this study supports the use of fine scale resolution maps based upon UAV-derived estimations of pasture biomass to guide grazing management decisions. Further, this work also demonstrates that it is technically possible to link UAV data and process-based crop models for use as a decision support tool to monitor, assess and plan grazing management. However, to become a practical approach for use by land managers and farmers future technical work must focus on the automation of UAV data processing and mapping, and pasture modeling steps to allow for rapid output data that can be available near real-time.

## Supporting information

S1 Table Summary of statistics indicating SALUS model performance for herbage mass accumulation (kg DM ha^-1^) of tall fescue and ryegrass regrowths from different residual pasture biomass in spring and summer of Exp. 1. (PDF)

S2 Table MDP model parameters for tall fescue and ryegrass pastures. (PDF)

S1 Data (XLS)
