# Supplementary material for: Estimation of spatial and temporal variability of pasture growth and digestibility in grazing rotations coupling unmanned aerial vehicle (UAV) with crop simulation models
Source: PLoS One. 2019 Mar 13;14(3):e0212773. doi: 10.1371/journal.pone.0212773 (PMC6415791; doi:10.1371/journal.pone.0212773)
Supplement: S1 Table — (PDF) [file pone.0212773.s001.pdf]

S1 Table. Summary of statistics indicating SALUS model performance for herbage mass accumulation (kg DM ha^{-1}) of tall fescue and ryegrass regrowths from different residual pasture biomass in spring and summer of Exp. 1.

	All	Spring	Summer	Tall fescue	Ryegrass
n	72	30	42	39	33
Actual mean	2722	2974	2542	2668	2785
Actual SD	1233	1446	1037	1157	1334
Simulated Mean	2511	2623	2432	2344	2709
Mean bias	211	352	110	325	76
RSME	506	501	509	590	390
Mean Prediction Error (%)	19	17	20	22	14
R^2	0.89	0.97	0.80	0.86	0.93
Pearson Correlation Coefficient - r	0.94	0.98	0.89	0.93	0.96
