# Supplementary material for: Estimation of spatial and temporal variability of pasture growth and digestibility in grazing rotations coupling unmanned aerial vehicle (UAV) with crop simulation models
Source: PLoS One. 2019 Mar 13;14(3):e0212773. doi: 10.1371/journal.pone.0212773 (PMC6415791; doi:10.1371/journal.pone.0212773)
Supplement: S2 Table — (PDF) [file pone.0212773.s002.pdf]

S2 Table. MDP Model parameters for tall fescue and ryegrass pastures.

Symbol	Parameters	Unit	Fescue	Ryegrass
Tb	Base temperature	ºC	4	=
LLS	Leaf Lifespan	ºCd	630	330
α	Length sheath fraction of residual height	cm cm^{-1}	0.71*	=
SER	Sheath elongation rate	cm ºCd^{-1}	0.0081*	=
ʊ	Sheath length effect on LER	cm cm^{-1}ºCd^{-1}	0.009*	x2
LAi	*a* and *b* in Eqn.3	-	As given in text*	x2
L_{NDF}	Length effect on NDF	% cm^{-1}	0.106	=
L_{NDFD}	Length effect on NDFD	% cm^{-1}	-0.529	=
$\delta_{NDFD\text{-}G}$	Rate of NDFD decline in LLS	% °Cd^{-1}	-0.0398	=
$\delta_{NDFD\text{-}S}$	Rate of NDFD decline in senescence	% °Cd^{-1}	-0.1206	=
NDF_0	Minimum NDF leaf blade	%	51.5	46.5
$NDFD_0$	Maximum NDFD leaf blade	%	70.1	90.1
NDF_S	NDF of senescent leaf blade tissues	%	64.9	=

* Calculated from Insua et al. (2017)
